# 

**Published:** 1914-03-21

**Authors:** 


### CONTENTS.

| | PAGE |
|---|---|
| **A Radium Policy for Poor-Law Unions** ... | 661 |
| **The Humbug of Closing Beds** ... ... ... ... | 662 |
| **Hospital and Institutional News** ... ... ... | 663 |

Radium and the Canadian Government—Isolation Hospital Flooded Out—Molière on the Nursing Home—A Hospital Strike in Rome—The Reconstruction of Brownlow Hill Workhouse—The Future Poor-Law Unit—A Poison Museum—The County Council and Mental Deficiency—The Parent of Nursing Homes—A Medical Officer's Claim to a Pension—Lord Wemyss on Homœopathy — Deterrents from Sanatorium Treatment—Syphilis Departments at General Hospitals—Cambridge Medical School and State Aid—Trade Tribute to the Middlesex Hospital—A Course in Tropical Hygiene—The Insurance Act and "Extravagant Prescribing"—Proposed New Hospital for Dundee—Mental Defectives in Victoria—The Sub-division of Hospital Work—Our Prize Scheme for Equipment "Tips"—The Supply of Cod-liver Oil—This Week's Drug Market—To Our Readers.

[*See over.*]

### CONTENTS—(*continued*).

PAGE

**Radium and Radio-Activity** ... ... ... 669
A Survey of its Therapy and Finance.
The Therapeutic Applications of Radiations.
Cost, and the Varieties of Radiations.
Application and Dosage.
Dangers and Reactions of Radium Treatment.
The Results of Radium Therapy.

**The Doctors' Wife** ... ... ... ... ... ... 675
The Doctors' Wives Association: A Free Service and Purchasing Agency.
The First Essential to Successful Co-operation.
The Purchasing Power of a Sovereign—Co-operation—The First Step to Success —An Independent Organisation—How Help May be Given—A Bureau of Information.

**Hospital Treatment and National Insurance...** 681
Anniversary Meeting at Leicester Royal Infirmary.

PAGE

**Sir Arthur Downes on Poor-Law Progress ...** 684
New Theatre at Tynemouth Union.

**The Editor's Letter-Box** ... ... ... ... 685

Medico-Psychology—Consultants and Press Announcements.

**The Mental Hospital Service** ... ... ... 686

**Round the World** ... ... ... ... ... 687

Fellow-Workers and their Doings.

**Institutional Needs** ... ... ... ... ... 688
A Provincial Centre for Hospital Furniture—"Hero" Liqueur Whisky—The "Eureka" Bandage — Invalid Lifters — "Regulin"—Institutional Waste Disposal.

**The National Medical Union (Official) ...** ... 690

**The Institutional Worker** ... ... (Suppl.) 1
II.—Service of Food in Nursing Institutions.
Questions for March.
Questions Invited.

